# Time-related changes in the knowledge of HIV/AIDS among followers of various religions in India

**DOI:** 10.12688/f1000research.133585.2

**Published:** 2023-11-15

**Authors:** Amna Khalid, Rizwan Qaisar, Firdos Ahmad, M. Azhar Hussain, Asima Karim

**Affiliations:** 1 Health Promotion Research Group, Research Institute of Medical and Health Sciences, University of Sharjah, Sharjah, Sharjah, 27272, United Arab Emirates; 2 Department of Family and Community Medicine and Behavioral Sciences, College of Medicine, University of Sharjah, Sharjah, 27272, United Arab Emirates; 3 Cardiovascular Research Group, Research Institute of Medical and Health Sciences, University of Sharjah, Sharjah, 27272, United Arab Emirates; 4 Basic Medical Sciences, College of Medicine, University of Sharjah, Sharjah, 27272, United Arab Emirates; 5 Department of Biomedical Sciences, College of Health Sciences, Abu Dhabi University, Abu Dhabi, 59911, United Arab Emirates; 6 Department of Finance and Economics, College of Business Administration, University of Sharjah, Sharjah, 27272, United Arab Emirates; 7 Department of Social Sciences and Business, Roskilde University, Roskilde, Region Zealand, DK-4000, Denmark; 8 Iron Biology Research Group, Research Institute of Medical and Health Sciences, University of Sharjah, Sharjah, 27272, United Arab Emirates

**Keywords:** HIV, AIDS, knowledge, religions, socio-demographic

## Abstract

**Background:**

The public knowledge levels about Human Immunodeficiency-Virus/Acquired Immunodeficiency Syndrome (HIV/AIDS) have been assessed in previous studies; however, time-related trends in association with socio-demographic standards among the followers of major religions in India are not known.

**Objectives:**

We assessed the 2005-06, 2015-16, and 2019-21 demographic and health survey (DHS) data from India to investigate trends in the levels of knowledge of HIV/AIDS among Hindus, Muslims, and Christians in relation to standard socio-demographic variables over a period of 16 years.

**Methods:**

The age range of the population was 15-54 years (n=611,821). The HIV/AIDS-related knowledge was assessed by developing a composite index based on ten questions about several aspects of HIV/AIDS, such as the mode of spread. We applied Chi-square and Kruskal-Wallis tests to investigate whether people had heard about HIV/AIDS and their overall HIV knowledge in relation to several socio-demographic standards.

**Results:**

Generally, a higher increase in knowledge level was found between the first and second DHS surveys (2006-2016) as compared to between the second and third DHS surveys (2016-2021). We found the highest increase in the level of HIV/AIDS knowledge among Christian women followed by Hindus, whereas Muslims had the least increase over 16 years. Being a female, uneducated, poor, previously married, or having rural residence were associated with the highest increase in the knowledge of HIV/AIDS.

**Conclusion:**

Christian women had the highest increase in HIV/AIDS-related knowledge then came Christian men and followers of other religions. We also found the highest increase in HIV/AIDS-related knowledge among the poorest, uneducated, and rural residents. Our findings may help formulate public health strategies targeting various less knowledgeable groups to reduce the incidence of HIV/AIDS.

## Introduction

India has the third largest proportion of patients infected with Human Immunodeficiency-Virus/Acquired Immunodeficiency Syndrome (HIV/AIDS) in the world. ^1^ The primary way to fight against this highly infectious disease is to increase knowledge and awareness and modify common people’s behaviour. Poor knowledge about the disease, lack of awareness about its modes of transmission, and having negative perceptions about HIV/AIDS-positive people can affect the preventive programs to control the spread of HIV/AIDS. India is a multicultural country having residents of various cultural beliefs, varying education levels, and several other population dynamics have been reported to affect an individual’s knowledge of HIV/AIDS in India. ^1^

In the past decade, HIV/AIDS knowledge and attitudes have been quite extensively explored. ^2^ Several factors such as education level, wealth status, caste, residential status, media exposure have been considered to significantly affect the level of knowledge about HIV/AIDS in India. ^3^ Religion is a crucial factor having a widespread effect on the health practices of an individual. The influence of religious organizations is well recognized in the fight against this deadly epidemic. ^4^ Furthermore, sexual health awareness, a critical protector against the spread of HIV/AIDS, may differ among followers of different religions. ^5^ Thus, engagement in unsafe sexual practices such as unprotected sex, extramarital relationships, considered potent factors in the spread of this disease, markedly could depend on the religious beliefs of a person. ^5^ Research available on religion and HIV/AIDS mostly emphasizes on the role of religion as a resource for people living with HIV/AIDS and is useful in helping these people to survive and find a meaning of life. ^6^ Minimal research studies have been conducted to define the multifaceted effect of religion on knowledge related to HIV/AIDS. The distinct traditions of religious communities have various themes that are intertwined with varying emphases that impact the practices of those communities. These practices and beliefs have often led to public pronouncements on HIV/AIDS education, prevention, and care, as well as to the shaping of public attitudes toward those afflicted by or at risk of HIV infection. ^7^ Therefore, it is important to explore the attitudes towards HIV/AIDS across religions to design effective policies to curb the epidemic of HIV/AIDS.

HIV/AIDS incidence in India had an estimated peak level of 0.54% in 2000-2001. However, the prevalence of HIV declined to 0.33% in 2010 and 0.22% in 2020. ^8^ Additionally, HIV/AIDS-related mortality rates in India have dropped by 82% since 2010. ^7^ These alterations can be attributed to changes in the background socio-demographic factors. Life in the rural communities is changing with the increase in educational opportunities, employment, exposure to mass media, wealth, and widespread use of contraceptives. ^9^ Despite these changes, society is still conservative and traditional in most parts of India. Although there is an overall increase in the awareness of HIV/AIDS preventive methods and modes of transmission, religious beliefs may still hinder this process. For example, age at marriage is still quite low for women, sex education and spreading of information about contraception are still not widely accepted. We have very limited data available about trends in HIV/AIDS knowledge among followers of various religions. The scarce literature on this topic involves homogenous data with indefinite conclusions, thereby emphasizing the importance of conducting a comprehensive study with a large representative sample to obtain more solid and generally applicable results.

We aimed to fill these gaps by investigating the trends of HIV/AIDS knowledge over time among major religions including Hindus, Muslims, and Christians, of India. We hypothesized that: 1) knowledge/understanding of HIV/AIDS has significantly increased with time among Hindus, Muslims, and Christians; 2) Sex-specific differences exist, with males being more knowledgeable than females; 3) Several socio-demographic factors such as age, residential status, wealth status, marital status, and level of education can affect the trend in knowledge of HIV/AIDS among followers of three religions. To test these hypotheses, we assessed data from three Demographic and Health Surveys (DHS 2005-2006; 2015-2016; 2019-2021) of India in a retrospective manner.

## Methods

The data utilized in this paper belongs to the DHS conducted during 2005-06, 2015-16, and 2019-21. DHS is a representative harmonized cross-sectional survey comprising almost all less developed countries around the world often covering several years. ^10^ Since this is a representative survey of the whole of the Indian population, both few people with HIV/AIDS as well as individuals without HIV/AIDS are included in the sample. The DHS collects data on different public health topics, socio-economic factors, living conditions, as well as demographics. This enables drawing a more complete picture of countries’ different vulnerable sub-populations.

The surveys used for India was conducted in two phases during each round. For the first survey, the first-phase data collection was carried out between November 2005 and May 2006, and the second-phase data collection was carried out between April and August 2006. For the second survey, the data collection was done from 20 ^th^ January 2015 to 4 ^th^ December 2016. For the last survey, first-phase data were collected from 17 ^th^ June 2019 to 30 ^th^ January 2020 while second-phase data were collected from 2 ^nd^ January 2020 to 30 ^th^ April 2021.

The questionnaires behind our applied data were the individual questionnaires for women and the questionnaire for men. ^9^^,^^11^^,^^12^ According to the published country reports for India, the sample size related to knowledge about HIV/AIDS for the three DHS surveys were 198,754, 224,531, and 201,158 respectively, which is 624,443 in total with 353,519 women and 270,924 men. In the actual accessed data sets applied here (IAIR52FL, IAIR74FL, IAIR7AFL, IAMR52FL, IAMR74FL, and IAMR7AFL), we ended up with a final data set of 611,821 individuals (337,568 interviewed women and 274,253 interviewed men). Reported knowledge about HIV/AIDS in India according to the three surveys (unweighted average) was 74.5% for women and 88.7% for men, while in our analyses, the percentage with knowledge about HIV/AIDS was 74.7% for women and 88.5% for men. Thus, despite the discrepancy in sample sizes, HIV-related estimates published by IIPS (International Institute for Population Sciences) and ICF (Inner City Fund) and those replicated by us were very close with only negligible estimate differences, which meant we were confident about the reliability of the sample. The original sample size was even higher, but after excluding individuals with missing information for different variables, we ended up with the 611,821 respondents.

When estimating different parameters, data were weighted by applying household weights (variable v005 or sv005 for women and mv005 for men), e.g., we used population weights giving us estimates for the adult 15-49 years old population (men above aged 50-54 years are also included).

The variables included in measuring individual HIV/AIDS knowledge represented whether the respondent knew: about HIV; condom use reduces HIV risk; only having one uninfected partner can reduce the HIV risk; mosquito bites do not infect people with HIV; sharing food with a HIV infected person does not give HIV; that a healthy looking person can have been HIV infected; blood transfusion can lead to HIV infection; that injecting drugs can lead to HIV infection; existing drugs can prevent HIV transmission from mother to baby; and, existing drugs can prolong the life of HIV infected people. These ten questions were analysed separately ( Table 1), and the same ten questions are also transformed into a single composite index using principal component analysis (PCA). ^13^ Thus, the composite index is not directly taken from the DHS, but on the other hand it is based on DHS variables. The Cronbach Alpha was in the very acceptable range (0.86) and the eigenvalue of the first component was 4.70, for the second component 1.22, while the third component had an eigenvalue of 0.10. The first component represented 47% of the variation in the ten HIV knowledge indicators, while the second component only added another 12%. We, therefore, decided to continue with only one principal component as a composite index representation of the complex phenomena called HIV/AIDS knowledge. The PCA composite index produced from the stata12 statistical software varied between -3.69 and 2.94 and was therefore transformed linearly to take values between 0 and 100. Since the scale of this composite measure has no standardized unit, we used the term point(s) to define it, implying that more points indicate higher overall HIV knowledge. Although the more technical principal component analysis procedure was applied, the interpretation of the composite index was straight forward in this case since its correlation with a simple average of the ten questions (where a true answer was coded as 1 and a wrong answer was coded as 0) was a staggering r=0.998. The correlation with the ten questions as well as the average of the ten question answers are shown in Table 2, where the correlations between the composite index and the ten questions were between 0.50 and 0.81.

**Table 1.** The individual components of HIV knowledge indicators.

No	Phrasing in the paper	Full phrasing in the questionnaire	Answers
1	Heard about HIV/AIDS	Have you ever heard of HIV?	Yes, No
2	Condom use protects	Can people reduce their chances of getting HIV/AIDS by using a condom every time they have sex?	Yes, No, Don't Know
3	Only one uninfected partner	Can people reduce their chances of getting HIV/AIDS by having just one uninfected sex partner who has no other sex partners?	Yes, No, Don't Know
4	Mosquito bites	Can people get HIV/AIDS from mosquito bites?	Yes, No, Don't Know
5	Sharing food	Can people get HIV/AIDS by sharing food with a person who has AIDS?	Yes, No, Don't Know
6	Healthy looking person	Is it possible for a healthy-looking person to have HIV/AIDS?	Yes, No, Don't Know
7	Blood transfusion	Can people get HIV/AIDS by blood products or blood transfusion?	Yes, No, Don't Know
8	Injecting drugs	Can people get HIV/AIDS by injecting drugs?	Yes, No, Don't Know
9	Transmission from mother to baby	Are there any special medications that a doctor or a nurse can give to a woman infected with HIV/AIDS to reduce the risk of transmitting HIV/AIDS to the baby?	Yes, No, Don't Know
10	Prolong HIV infected person's life	Have you heard about special antiretroviral drugs (USE LOCAL NAME(S)) that people infected with HIV/AIDS can get from a doctor or a nurse to help them live longer?	Yes, No, Don't Know

**Table 2.** Distribution of the studied population (n=611,821) by socio-demographic characteristics, separately for religions and years.

Religion	Variable	Category	2005-06	2015-16	2019-21	N
Hindu	Sex	Males	37.8	48.2	48.7	215482
		Females	62.2	51.8	51.3	261074
	Age	15-29 years	51.3	48.6	46.8	232720
		30-54 years	48.7	51.4	53.2	243836
	Residence	Urban	46.6	29.2	24.4	156688
		Rural	53.4	70.8	75.6	319868
	Marital status	Married	68.6	68.4	67.5	324658
		Previously married	3.4	2.9	3.0	14621
		Never married	28.1	28.8	29.5	137277
	Wealth	Poorest 20%	11.9	18.7	20.4	81903
		Middle 60%	57.5	61.8	62.8	289780
		Richest 20%	30.7	19.5	16.8	104873
	Education	None	25.5	20.4	17.6	100259
		Primary or secondary	61.4	65.2	65.8	306231
		Higher	13.1	14.4	16.6	70066
Muslim	Sex	Males	36.4	45.9	46.7	37127
		Females	63.6	54.1	53.3	48719
	Age	15-29 years	57.8	53.8	51.8	46720
		30-54 years	42.2	46.2	48.2	39126
	Residence	Urban	59.1	41.6	34.6	38500
		Rural	40.9	58.4	65.4	47346
	Marital status	Married	64.4	63.0	64.7	54861
		Previously married	3.1	2.2	2.2	2112
		Never married	32.5	34.8	33.2	28873
	Wealth	Poorest 20%	9.1	12.7	16.7	10999
		Middle 60%	63.3	67.2	66.2	56414
		Richest 20%	27.6	20.1	17.1	18433
	Education	None	34.0	25.7	22.2	23324
		Primary or secondary	59.3	64.4	66.1	54396
		Higher	6.7	9.9	11.7	8126
Christian	Sex	Males	37.7	46.6	47.8	21644
		Females	62.3	53.4	52.2	27775
	Age	15-29 years	52.9	46.5	44.9	23857
		30-54 years	47.1	53.5	55.1	25562
	Residence	Urban	44.2	32.2	23.2	16647
		Rural	55.8	67.8	76.8	32772
	Marital status	Married	57.8	61.7	62.1	29864
		Previously married	4.2	4.3	4.1	2077
		Never married	38.0	34.0	33.8	17478
	Wealth	Poorest 20%	5.2	11.5	24.7	6586
		Middle 60%	63.2	71.5	66.1	33059
		Richest 20%	31.5	17.0	9.2	9774
	Education	None	14.2	12.5	13.1	6559
		Primary or secondary	72.9	74.4	72.5	36217
		Higher	12.9	13.1	14.4	6643
Total			100	100	100	611821

Source: Own calculations based on DHS for India from the years 2005-06, 2015-16, and 2019-21.

We expected differences between different socio-demographic groups’ HIV knowledge, and therefore statistical tests were carried out to investigate inter-group differences regarding the composite HIV knowledge index. The traditional T test was not used since the index was not normally distributed. Instead, we relied on the Kruskall-Wallis rank test (on unweighted data). ^14^ In the case of significance of differences in the percentage of the population who heard about HIV/AIDS, we relied on the Chi-square test (also on unweighted data).

The central religion variable recorded individual faith and we included here only the major religions due to sample size considerations. In addition, we included important socio-economic and demographic variables ( Table 2). There were 611,821 respondents in total. Among the respondents, 476,556 belonged to Hinduism, 85,846 followed Islam, and 49,419 believed in Christianity. The other major religion, Sikhism, was also investigated, but both the officially DHS reported estimates and our own estimates saw an unexpected dip in HIV knowledge for this group in the middle survey, which was not explained anywhere, and therefore this religion was excluded from the analysis. The sex distribution within religion was almost the same in each of the surveys. A similar pattern was observed for the age distribution, where around half of the participants were between 15 and 29 years of age, and the other half were between 30-49 years age (30-54 years for males). Generally, the sample was skewed towards being from rural areas. Around 2/3 of the interviewed Hindus were married, while this fraction was a little lower among Muslims, and even lower among Christians. The distribution along wealth quintiles largely followed the proportions of populations in each religion, though Hindus and Muslims tended to be more often in the top quintile compared to Christians. Christians were least frequently in the no education category, while Muslims least frequently were in the higher education category (74%).

## Results

**Figure 1.**
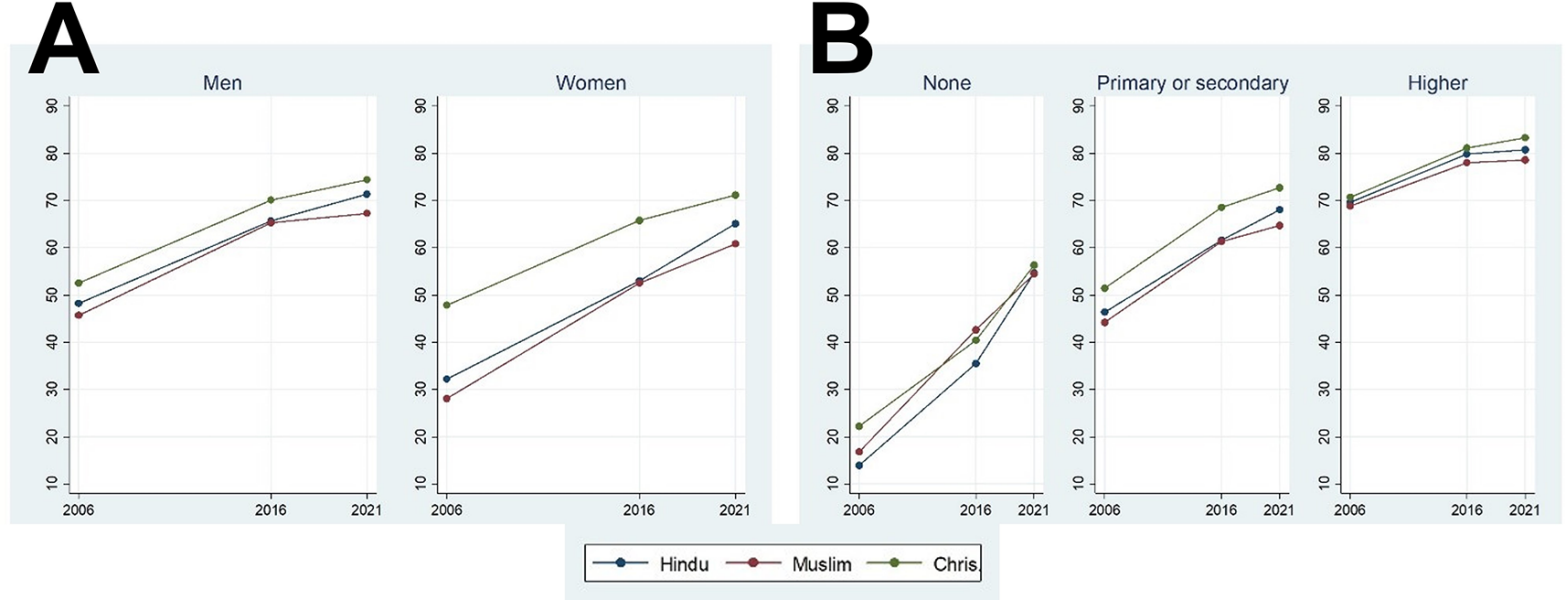
Average HIV knowledge index of participants of different sexes (A) and education levels (B) among Muslims, Hindus, and Christians of India over a time course of 16 years at three time points.

The development in percentage of the Indians who have heard about HIV/AIDS is presented in Figure 1. Fortunately, over time there has been a remarkable increase in the fraction of people who heard about the disease. This positive development was seen regardless of religion, sex, and educational status. There was a higher spread of HIV knowledge among Christians compared to the followers of two other religions (except for people without education in 2015-16). Muslims had slightly lower prevalence of HIV knowledge compared to Hindus (except among people without education). Finally, we generally observed that the speed of increased HIV knowledge was generally highest from the first to the second survey (from 2005-06 to 2015-16) compared to from the second to the third survey (2019-21), except for people without education, which had a much lower initial level of prevalence.

Table 3 represents a broader view by employing the composite HIV knowledge index and by further looking into different socio-demographic groups of the population. We observed an increase in HIV knowledge for every presented population sub-group, as the HIV index increased from 38.0 to 59.6 to 67.6 points over the analyzed 16-year-time period. This represented a 78% increase from 2005 to 2019/21. The highest increase was recorded for people without education, where average HIV knowledge increased from 15 to 55 representing a 276% increase. A greater increase in HIV knowledge was also seen for the bottom quintile (265%), previously married (130%), rural residents (109%), and females (102%). The lowest increase in overall HIV knowledge was seen for highly educated people whose average increased from 69.6 to 80.6 (a 16% increase), and low increases were also seen for the richest quintile (30%) and never married people (41%). We observed a clear tendency that the lower the initial HIV knowledge in 2005-06, the higher is the increase in HIV knowledge from the first to the last survey, which meant that differences in the level of HIV knowledge between different population groups had been reduced over time. These results corroborate that India’s response to HIV/AIDS using targeted interventions was successful in raising awareness about the epidemic which is well documented. ^15^^–^^17^ The task now lies in sustaining success due to the emergence of newer pandemics like COVID-19.

**Table 3.** Average HIV knowledge index points according to socio.

Variable	Category	2005-06	2015-16	2019-21	N	P value
Sex	Males	48.0	65.8	70.8	274253	<0.0001
	Females	32.0	53.9	64.7	337568	<0.0001
Age	15-29 years	40.6	60.3	67.0	303297	<0.0001
	30-54 years	35.2	58.9	68.2	308524	<0.0001
Residence	Urban	51.8	68.6	73.6	211835	<0.0001
	Rural	30.9	54.5	64.6	399986	<0.0001
Marital status	Married	35.0	58.5	67.6	409383	<0.0001
	Previously married	26.5	51.8	63.2	18810	<0.0001
	Never married	48.1	62.7	68.0	183628	<0.0001
Wealth	Poorest 20%	15.1	38.3	55.1	99488	<0.0001
	Middle 60%	36.5	59.6	68.0	379253	<0.0001
	Richest 20%	59.9	74.9	78.0	133080	<0.0001
Education	None	14.5	37.0	54.6	130142	<0.0001
	Primary or secondary	46.2	61.9	67.7	396844	<0.0001
	Higher	69.6	79.7	80.6	84835	<0.0001
Total		38.0	59.6	67.6	611821	<0.0001

Note: Kruskal-Wallis tests were used to see whether there was independence over time. The test is carried out for each level of the socio-demographic variable.

Source: See Table 2.

We next investigated the individual components of HIV knowledge in the studied population ( Table 4). Each of the ten dimensions behind the HIV knowledge index showed a positive development from 2005-06 to 2019-21. The largest increases were seen for knowledge about injecting drugs can cause HIV as well as blood transfusions can cause HIV. On the other hand, there was only a modest increase in knowledge about the fact that sharing food does not lead to HIV. Notably, these developments were very similar across the followers of the three religions. Additionally, the development in all ten HIV knowledge indicators were similar for Hindus and Muslims and generally at a higher level than for Christians, which reflected that HIV knowledge among Christians were already at a higher level initially (2005-06) than for the two other religions. Although progress was registered for each of the ten indicators, the knowledge about the medications prolonging the lifespan of HIV infected people, medicine preventing HIV transmission from mother to baby, and sharing food as a potential mean of transmitting HIV was poor.

**Table 4.** Percentage with correct HIV knowledge (n=611,821) within ten dimensions across religions and time.

Religion	Question	2005-06	2015-16	2019-21	P value
Hindu (N=476556)	Heard about HIV/AIDS	69.0	81.2	90.7	<0.0001
	Condom use protects	48.7	65.4	75.2	<0.0001
	Only one uninfected partner	54.8	64.8	74.7	<0.0001
	Mosquito bites	43.6	56.3	59.2	<0.0001
	Sharing food	48.8	53.6	52.1	<0.0001
	Healthy looking person	46.1	59.1	69.1	<0.0001
	Blood transfusion	10.1	66.7	76.1	<0.0001
	Injecting drugs	7.0	64.4	73.7	<0.0001
	Transmission from mother to baby	19.5	35.8	49.3	<0.0001
	Prolong HIV infected person's life	10.5	22.6	35.3	<0.0001
Muslim (N=85846)	Heard about HIV/AIDS	64.6	81.6	88.2	<0.0001
	Condom use protects	43.4	64.2	71.3	<0.0001
	Only one uninfected partner	50.3	64.9	69.1	<0.0001
	Mosquito bites	37.1	49.8	53.4	<0.0001
	Sharing food	43.2	47.5	45.5	<0.0001
	Healthy looking person	42.3	61.9	68.2	<0.0001
	Blood transfusion	8.2	67.2	72.2	<0.0001
	Injecting drugs	5.6	63.7	69.3	<0.0001
	Transmission from mother to baby	15.1	34.0	44.1	<0.0001
	Prolong HIV infected person's life	8.2	20.6	32.9	<0.0001
Christian (N=49419)	Heard about HIV/AIDS	85.8	90.5	95.6	<0.0001
	Condom use protects	57.5	66.4	77.1	<0.0001
	Only one uninfected partner	67.2	66.0	76.6	<0.0001
	Mosquito bites	55.3	63.3	67.0	<0.0001
	Sharing food	64.7	67.8	65.7	<0.0001
	Healthy looking person	60.0	67.9	71.7	<0.0001
	Blood transfusion	17.2	76.9	83.9	<0.0001
	Injecting drugs	7.1	74.7	79.4	<0.0001
	Transmission from mother to baby	29.5	40.6	48.6	<0.0001
	Prolong HIV infected person's life	24.6	31.8	38.6	<0.0001

Note: Chi-square tests are used to test homogeneity over the three time periods.

Source: See Table 1 for details of questions.

The composite index of knowledge for various population subgroups is summarized in Table 5. The HIV knowledge markedly improved over time for the followers of all religions and for the subgroups of the population. The greatest increase in overall HIV knowledge was seen for Muslims in the bottom quintile of wealth, who saw an increase starting from 12.3 in 2005/06 to 39.4 in 2015/16 and ending with 53.5 in 2019/21, which implies an increase of 334% from 2005-06 to 2019-21. Large increases in HIV knowledge were also observed among Hindus without education (292%), poorest Hindus (259%), Muslims without education (226%), and the poorest Christians (165%). Least progress was made by Muslims with higher education, who exhibited an overall HIV knowledge of 68.8 in 2005/06 to 78.0 in 2015/16 to 78.6 in 2019/21, which meant an increase of only 14% from 2005-06 to 2019-21. Other low HIV knowledge increases over the period were seen for Hindus with high education (16%), Christians with high education (18%), richest Christians (25%), and the richest Hindus (30%). Moreover, in general, there was a high tendency that overall HIV knowledge increase over time was greater when baseline levels were low.

**Table 5.** Average HIV knowledge index points according to different religions and sociodemographic groups (n=611,821).

Religion	Variable	Category	2005-06	2015-16	2019-21	N	P value
Hindu	Sex	Males	48.2	65.7	71.3	215482	<0.0001
		Females	32.2	53.0	65.1	261074	<0.0001
	Age	15-29 years	41.1	60.3	67.6	232720	<0.0001
		30-54 years	35.2	58.5	68.5	243836	<0.0001
	Residence	Urban	52.5	69.2	74.5	156688	<0.0001
		Rural	31.4	54.4	65.1	319868	<0.0001
	Marital status	Married	35.1	58.4	68.1	324658	<0.0001
		Previously married	26.6	50.2	64.1	14621	<0.0001
		Never married	49.1	62.8	68.5	137277	<0.0001
	Wealth	Poorest 20%	15.4	38.1	55.3	81903	<0.0001
		Middle 60%	36.8	59.5	68.5	289780	<0.0001
		Richest 20%	60.3	75.5	78.5	104873	<0.0001
	Education	None	14.0	35.5	54.6	100259	<0.0001
		Primary or secondary	46.3	61.6	68.1	306231	<0.0001
		Higher	69.6	79.9	80.7	70066	<0.0001
Muslim	Sex	Males	45.7	65.3	67.3	37127	<0.0001
		Females	28.0	52.5	60.8	48719	<0.0001
	Age	15-29 years	36.1	58.3	63.0	46720	<0.0001
		30-54 years	31.8	58.1	65.2	39126	<0.0001
	Residence	Urban	46.8	64.0	68.8	38500	<0.0001
		Rural	25.4	53.1	60.8	47346	<0.0001
	Marital status	Married	31.7	57.4	64.2	54861	<0.0001
		Previously married	22.5	48.6	56.8	2112	<0.0001
		Never married	41.8	60.2	64.4	28873	<0.0001
	Wealth	Poorest 20%	12.3	39.4	53.5	10999	<0.0001
		Middle 60%	33.1	58.0	64.3	56414	<0.0001
		Richest 20%	55.9	70.1	74.0	18433	<0.0001
	Education	None	16.7	42.6	54.5	23324	<0.0001
		Primary or secondary	44.2	61.3	64.8	54396	<0.0001
		Higher	68.8	78.0	78.6	8126	<0.0001
Christian	Sex	Males	52.5	70.1	74.4	21644	<0.0001
		Females	47.8	65.8	71.2	27775	<0.0001
	Age	15-29 years	49.6	67.4	72.9	23857	<0.0001
		30-54 years	49.4	66.2	72.8	25562	<0.0001
	Residence	Urban	59.7	76.2	79.2	16647	<0.0001
		Rural	41.7	60.3	69.5	32772	<0.0001
	Marital status	Married	48.7	65.0	72.7	29864	<0.0001
		Previously married	38.0	69.3	60.7	2077	<0.0001
		Never married	52.8	69.8	74.7	17478	<0.0001
	Wealth	Poorest 20%	21.8	39.1	57.8	6586	<0.0001
		Middle 60%	45.9	65.9	73.2	33059	<0.0001
		Richest 20%	64.6	80.1	80.8	9774	<0.0001
	Education	None	22.2	40.4	56.3	6559	<0.0001
		Primary or secondary	51.4	68.6	72.7	36217	<0.0001
		Higher	70.6	81.1	83.3	6643	<0.0001

Note: Kruskal-Wallis tests were used to see whether there was independence over time. The test is carried out for each level of the socio-demographic variable.

Source: See Table 2.

## Discussion

To our best knowledge, awareness trends of HIV/AIDS among followers of different religions over a period of 16-years has been assessed for the first time by using the most up-to-date data. We found that Christians had the highest increase in knowledge followed by Hindus, whereas Muslims had the least increase. An increase in the HIV/AIDS knowledge was higher between the first and second DHS surveys (2006-2016) as compared to the second and third DHS surveys (2016-2021). We report that overall, there was a 78% increase in HIV/AIDS knowledge in the last 16 years, where men were more knowledgeable than women, and higher education level was associated with more HIV/AIDS-related knowledge, irrespective of the religion of the participants. After applying the HIV knowledge index comprising ten key questions related to HIV/AIDS awareness, we found that the highest increase was observed among females, non-educated, poorest, previously married, and rural residents. Looking at the individual questions of the HIV knowledge index, the highest increase was observed in the knowledge about injecting drugs and lowest increase in the knowledge about sharing food. This study also revealed that although HIV/AIDS knowledge improved for the participants of all religions with time, residential area-associated, education-associated, and wealth-associated disparities in knowledge increase remained large.

We found that although overall knowledge of HIV/AIDS increased significantly over the studied time, it still needs improvement, as approximately one-third of the overall population reported lack of awareness about HIV/AIDS-related knowledge. Our findings indicate that there was an overall increase in HIV/AIDS-related knowledge over time regardless of religion, sex, and educational status. This can be attributed to the fact that there has been an overall increase in HIV/AIDS awareness through educational campaigns introduced by both governmental and non-governmental organizations. We also found that the trend in HIV-related knowledge was substantially influenced by background socio-demographic factors. We found that women had a higher trend of increase in HIV-related knowledge as compared to men, consistent with already reported findings. ^18^ This could be because women have a greater frequency of listening to radio or watching television. However, men tend to more often busy at their work and their sources of information are thus limited.

Among the followers of included religions, Christians reported the highest increase in the level of HIV/AIDS-related knowledge, whereas Muslims were found to be the least in this regard. In this study, individuals belonging to the Christian community, which constitutes a relatively small religious minority, demonstrated a more comprehensive understanding of HIV/AIDS. This could be attributed to their higher representation in North-Eastern India, an area with a heightened prevalence of HIV. This implies that the local administration, along with sociocultural and religious organizations, have played an effective role in advancing HIV education within these communities. ^3^ Similarly, previous studies also reported that Christians demonstrated higher knowledge of HIV/AIDS than other religions. ^19^^,^^20^ These findings can be described in the light of data released on education level of religious communities by the government in the last decennial population census of India, where the literacy rate of Christians was reported to be 74%, Hindus had 64%, and Muslims had 57%. Educational attainment level and HIV/AIDS knowledge have a positive correlation and play a vital role in reducing the transmission of disease through increasing awareness. ^21^^,^^22^ More educated people are more likely to be aware of the effective preventive strategies of HIV/AIDS, tending to be more aware and show more adherence to healthy behaviors, which are critical components for HIV/AIDS prevention. However, an interesting finding in our study was in terms of the highest increase over time in HIV-related knowledge that was observed amongst the illiterate participants. This can be ascribed to the widespread mass media usage in the world generally and in the developing countries in particular. ^23^^,^^24^

Residence, wealth, and marital status are highly correlated and impactful variables regarding HIV/AIDS-related knowledge in a community. In our results, wealth status appears to be highly correlated with HIV comprehensive knowledge. In the three surveys analyzed in this study, irrespective of the religious beliefs, participants in the lowest quintile had the least HIV/AIDS knowledge. In the most recent DHS (2019-2021), half of the study participants in the lowest wealth quintile (55.1%) had HIV/AIDS-related knowledge. At the same time, awareness about HIV/AIDS was rather prevalent among people in the highest wealth quintile (78%). These findings are in line with the previously reported general trend, where the level of knowledge of HIV/AIDS is significantly higher among wealthier as compared to the poorer. ^25^^,^^26^ This can be attributed to the fact that people with more wealth have more chances of exposure to modes through which HIV/AIDS-related knowledge is disseminated. At one point in time the DHS data revealed that the poorest participants had least HIV/AIDS awareness. However, the time related 16 years’ analysis of the DHS surveys done in our study revealed the first-ever comprehensive picture of the time-related trends in HIV/AIDS-related knowledge. Accordingly, our study showed the highest increase in knowledge among the poorest participants and vice versa.

A similar trend was found in other background socio-demographic standards such as non-educated, previously married participants, and rural residents, which showed highest increase in HIV/AIDS-related knowledge as compared to never married and urban residents. Moreover, the increase in knowledge was higher between the first and second DHS (between 2006-2016) as compared to the difference between the second and the last DHS (2016-2021). Our findings show a complex interaction of demographic standards in society with the time-related changes in HIV/AIDS-related awareness. These finding may reflect the importance of other factors such as mass media campaigns. ^23^ Moreover, there has been a substantial increase in internet users in India from 10.5% to 64.6%, during 2006 to 2019 and the increase is higher among rural as compared to urban dwellers. India’s rural internet users are growing much faster than urban residents as stated in a report based on internet adoption in India released in 2021. Similarly, the increasing trend of internet usage can possibly be the reason behind our findings that the non-educated Muslim and Hindu participants showed a significantly greater increase in HIV/AIDS-related knowledge as compared to their educated counterparts.

Our findings regarding the knowledge of participants concerning ten dimensions of spread of HIV/AIDS revealed the highest increase in knowledge about the use of injections and blood transfusions as the possible source of transmission of disease among the followers of all religions. These findings are consistent with previous studies, ascribed to the availability of television as main source of information. ^27^ Conversely, least increase in knowledge level was found about awareness that sharing food with the HIV/AIDS sufferers cannot be the source of spread of disease, which is consistent with previous studies. ^24^ Thus, HIV/AIDS-related stigma possibly still exists in India.

The findings of the study have many practical and clinical implications. Religious scholars and organizations can take positions, issue statements, and influence the consciences within their communities. These faith communities can participate in raising awareness about HIV/AIDS, offering free treatment, as well as promoting HIV/AIDS testing and preventative measures. The preventional and interventional programs may target the communities via their respective faith centres to ensure all have access to such programs. ^7^

The strength of the study is based on a nationally representative large dataset enhancing the reliability of our results. The simplicity of questions in the survey ensures the reliability of data irrespective of the educational status of the participant. This dataset is from a geographically similar population limiting the confounding factors such as ethnicity, race, etc. However, our study also has some limitations. We did not investigate factors, such as the potential influences of policy, sexual education in schools, government campaigns, and family on HIV knowledge in the study populations. We did not investigate the amount of religiosity and religious practice for a given follower. Therefore, our study does not consider the effects of the strength of belief on HIV knowledge.

We conclude that religious beliefs significantly affect the awareness of people about HIV/AIDS. We found that Christian men were significantly more knowledgeable of HIV/AIDS than their female counterparts and followers of other religions. We also found the highest increase in HIV/AIDS-related knowledge level among the poorest and non-educated participants over a 16 years’ period. Our findings may be helpful in designing strategies for public health interventions targeting a less knowledgeable cohort of participants.

## Ethical approval

Not required since it was secondary data.

## Data Availability

Data used in this study are from the IAHR74FL, IAIR74FL and IAMR74FL datasets for India from 2015-16 with face-to-face interviews available from the Demographic and Health Survey (DHS) website https://dhsprogram.com/data/dataset/India_Standard-DHS_2015.cfm?flag=0. Access to the dataset requires registration and is granted only for legitimate research purposes. A guide for how to apply for dataset access is available at https://dhsprogram.com/data/Access-Instructions.cfm.
