# Supplementary material for: Time-related changes in the knowledge of HIV/AIDS among followers of various religions in India
Source: F1000Res. 2023 Nov 15;12:460. Originally published 2023 May 2. [Version 2] doi: 10.12688/f1000research.133585.2 (PMC10682603; doi:10.12688/f1000research.133585.2)
Supplement: Peer review history

# Reviewer report: Reviewer response for version 2

**DOI:** 10.5256/f1000research.158034.r223164

**Published:** 2023-11-27

**Authors:** M Rifqi Rokhman

**Affiliations:** 1 Universitas Gadjah Mada, Yogyakarta, Special Region of Yogyakarta, Indonesia; 2 University of Groningen, Groningen, The Netherlands

I have reviewed the revised version, and the authors' revisions are sufficient for acceptance and indexing.

Is the work clearly and accurately presented and does it cite the current literature?

Yes

If applicable, is the statistical analysis and its interpretation appropriate?

Partly

Are all the source data underlying the results available to ensure full reproducibility?

Yes

Is the study design appropriate and is the work technically sound?

Yes

Are the conclusions drawn adequately supported by the results?

Partly

Are sufficient details of methods and analysis provided to allow replication by others?

Yes

Reviewer Expertise:

HIV knowledge, quality of life

I confirm that I have read this submission and believe that I have an appropriate level of expertise to confirm that it is of an acceptable scientific standard.

# Reviewer report: Reviewer response for version 2

**DOI:** 10.5256/f1000research.158034.r223163

**Published:** 2023-11-21

**Authors:** Angga Wilandika

**Affiliations:** 1 Universitas Aisyiyah Bandung, West Java, Indonesia

I appreciate the corrections and clarifications provided. I think the revisions made give a clearer picture of the study.

Is the work clearly and accurately presented and does it cite the current literature?

Yes

If applicable, is the statistical analysis and its interpretation appropriate?

Partly

Are all the source data underlying the results available to ensure full reproducibility?

Yes

Is the study design appropriate and is the work technically sound?

Yes

Are the conclusions drawn adequately supported by the results?

Partly

Are sufficient details of methods and analysis provided to allow replication by others?

Partly

Reviewer Expertise:

HIV/AIDS, Health literacy, Social stigma, Nursing, and Public health

I confirm that I have read this submission and believe that I have an appropriate level of expertise to confirm that it is of an acceptable scientific standard.

# Reviewer report: Reviewer response for version 1

**DOI:** 10.5256/f1000research.146586.r209608

**Published:** 2023-10-10

**Authors:** Angga Wilandika

**Affiliations:** 1 Universitas Aisyiyah Bandung, West Java, Indonesia

**Dear Author(s)**

 This manuscript discusses a pretty exciting topic, but the research focuses on differences in knowledge indices based on religion; this is quite sensitive and can lead to negative perceptions, prejudices and even stigmatisation of religion. Authors should, therefore, be more careful when reporting study results.

**INTRODUCTION**

- On page 3, paragraph 2, the Author writes: “Minimal research studies have been conducted to define the effect of religious beliefs on knowledge related to HIV/AIDS”. This study did not examine religious beliefs, but it did identify HIV knowledge among the majority religious community in India. Authors must distinguish between the concepts of "religious belief" and "religion", which are two very different concepts. Therefore, the introduction should explain why trends in HIV/AIDS knowledge among different religions should be studied.
- It should be considered that if the study results show a negative knowledge trend for one religious group, then the results of this study can stigmatise that religion. Please clarify this.
- Abbreviations must be explained. Please check the entire manuscript.

**METHODS**

- Please explain in more detail who is the sample in this study. Does the entire population involved in this study contain people with HIV, or are all people without HIV? This sample difference may affect the results regarding HIV/AIDS knowledge.
- On page 4, paragraph 3. The author writes: “These ten questions were analyzed separately (Table 1), and the same ten questions are also transformed into a single composite index using principal component analysis (PCA)”. PCA is a statistical technique for reducing the dimensionality of a dataset. Did you use the PCA technique in this study? Why did you use this technique? Isn't the data from the DHS? Please explain.

**DISCUSSION**

- Please add the practical and clinical implications (contributions) of the results of this study.
- In the discussion section, the author should discuss the role of religious organisations or religious leaders as one of the parties that can participate in HIV knowledge campaigns. Practical and clinical (contributions) of the results of this study.
- Check the discussion to ensure no religion is stigmatised due to the research.
- Please add a limitation study.

Is the work clearly and accurately presented and does it cite the current literature?

Yes

If applicable, is the statistical analysis and its interpretation appropriate?

Partly

Are all the source data underlying the results available to ensure full reproducibility?

Yes

Is the study design appropriate and is the work technically sound?

Yes

Are the conclusions drawn adequately supported by the results?

Partly

Are sufficient details of methods and analysis provided to allow replication by others?

Partly

Reviewer Expertise:

HIV/AIDS, Health literacy, Social stigma, Nursing, and Public health

I confirm that I have read this submission and believe that I have an appropriate level of expertise to confirm that it is of an acceptable scientific standard, however I have significant reservations, as outlined above.

# Response

**Published:** 2023-10-24

**Authors:** Asima Karim

**Affiliations:** Basic Medical Sciences, University of Sharjah, Sharjah, Sharjah, United Arab Emirates

We thank the reviewers for their time and for the favorable comments. We have addressed all the comments raised by the reviewer and highlighted the corrections throughout the revised manuscript. We believe that the reviewer's comments helped to improve the quality of the manuscript. Please find attached the revised version of our manuscript entitled **" Time-related changes in the knowledge of HIV/AIDS among followers of various religions in India". **The point-by-point response to the reviewer’s comments is provided below.

**Reviewer # 2**

**General comments:**

 This manuscript discusses a pretty exciting topic, but the research focuses on differences in knowledge indices based on religion; this is quite sensitive and can lead to negative perceptions, prejudices and even stigmatization of religion. Authors should, therefore, be more careful when reporting study results.

**Specific comments:**

**1.** **Introduction:**

 (A) On page 3, paragraph 2, the Author writes: “Minimal research studies have been conducted to define the effect of religious beliefs on knowledge related to HIV/AIDS”. This study did not examine religious beliefs, but it did identify HIV knowledge among the majority religious community in India. Authors must distinguish between the concepts of "religious belief" and "religion", which are two very different concepts. Therefore, the introduction should explain why trends in HIV/AIDS knowledge among different religions should be studied.

**Author response: **The suggested sentence has been modified accordingly in the revised manuscript. Furthermore, an explanation of why trends in HIV/AIDS knowledge among religions should be studied has been given. Please refer to the second paragraph of introduction section.

 (B) It should be considered that if the study results show a negative knowledge trend for one religious group, then the results of this study can stigmatize that religion. Please clarify this.

**Author response: **The purpose of the manuscript is to study the trends in HIV/AIDS knowledge over time among major religions including Hindus, Muslims, and Christians, in India. Therefore, we believe keeping these results does not stigmatize any religion as the trend across religions is upwards.

 (C) Abbreviations must be explained. Please check the entire manuscript.

**Author response: **This has been incorporated in the revised manuscript.

**2.** **Methods:**

 (A) Please explain in more detail who is the sample in this study. Does the entire population involved in this study contain people with HIV, or are all people without HIV? This sample difference may affect the results regarding HIV/AIDS knowledge.

**Author response:**

 The study contains both individuals with HIV and without HIV since this is a representative sample. We have now further clarified this in the revised manuscript.

 (B) On page 4, paragraph 3. The author writes: “These ten questions were analyzed separately (Table 1), and the same ten questions are also transformed into a single composite index using principal component analysis (PCA)”. PCA is a statistical technique for reducing the dimensionality of a dataset. Did you use the PCA technique in this study? Why did you use this technique? Isn't the data from the DHS? Please explain.

**Author response: **Yes, we did use the PCA technique in this study. We used it to measure the complex and latent concept of HIV knowledge. The included data is from the DHS, but our composite index, developed via PCA, is not directly from the DHS. Nevertheless, it is based on the listed ten questions from the DHS.

**3. Discussion:**

 (A) Please add the practical and clinical implications (contributions) of the results of this study.

**Author response: **This has been added in the 7 ^th^ paragraph of the discussion.

 (B) In the discussion section, the author should discuss the role of religious organisations or religious leaders as one of the parties that can participate in HIV knowledge campaigns. Practical and clinical (contributions) of the results of this study.

**Author response:** This has been added in the 7 ^th^ paragraph of the discussion.

 (C) Check the discussion to ensure no religion is stigmatised due to the research.

**Author response: **This has been cross-checked and we can assure that no religion is stigmatized.

 (D) Please add a limitation study.

**Author response: **Please refer to the second last paragraph of the discussion section.

# Reviewer report: Reviewer response for version 1

**DOI:** 10.5256/f1000research.146586.r202666

**Published:** 2023-09-27

**Authors:** M Rifqi Rokhman

**Affiliations:** 1 Universitas Gadjah Mada, Yogyakarta, Special Region of Yogyakarta, Indonesia; 2 University of Groningen, Groningen, The Netherlands

The study is interesting since HIV is one of the major public health concerns. Some revisions are required to improve the manuscript.

**Introduction:**

 "The scarce literature on this topic involves a small cohort with indefinite conclusions,  ...". What does 'a small cohort' refer to?

**Methods:**

 Please explain, how the authors' approach to evaluate whether the differences in HIV knowledge increase were mainly due to the difference based on religions but not due to differences in socio-demographic characteristics. Can the authors add an analysis about this?

**Results:**

 It is stated that 'The development in fraction of the Indians who have heard about HIV/AIDS is presented in Figure 1'. However, Figure 1 displays about average HIV knowledge. Please clarify.

 The discussions for Tables 3, 4, and 5 were mainly about the highest/lowest increase of HIV knowledge over a 16-year time period. If possible, please add a column providing the increase in HIV knowledge over this period in Tables 3, 4, and 5. Please also add subgroups based on religions in Table 3.

 "The greatest increase in overall HIV knowledge was seen for Muslims in the bottom quintile, ...". Is it based on wealth? If yes, please add this information to the sentence to make it clearer for the readers.

**Discussion:**

 "We found that Christians had the highest increase in knowledge followed by Hindus, whereas Muslims had the least increase." Did the authors mean by 'the highest increase' is actually 'the highest level of HIV knowledge'. Please clarify.

 "We observed a clear tendency that the lower the initial HIV knowledge in 2005-06, the higher is the increase in HIV knowledge from the first to the last survey, which meant that differences in the level of HIV knowledge between different population groups had been reduced over time." Please discuss this finding including the implication for public health interventions.

 "Christians reported the highest increase in the level of HIV/AIDS-related knowledge" then what are the implications of this study to help in designing strategies for public health interventions related to different religions? And again, does 'the highest increase' refer to 'the highest level' of HIV knowledge?

 "We found that men had a higher trend of increase in HIV-related knowledge as compared to females." However, in Christian, the HIV knowledge of men increased from 52.5 to 74.4 (an increase of 21.9), while women from 47.8 to 71.2 (an increase of 23.4). The same cases for Muslims and Hindus. Please clarify.

Is the work clearly and accurately presented and does it cite the current literature?

Yes

If applicable, is the statistical analysis and its interpretation appropriate?

Partly

Are all the source data underlying the results available to ensure full reproducibility?

Yes

Is the study design appropriate and is the work technically sound?

Yes

Are the conclusions drawn adequately supported by the results?

Partly

Are sufficient details of methods and analysis provided to allow replication by others?

Yes

Reviewer Expertise:

HIV knowledge, quality of life

I confirm that I have read this submission and believe that I have an appropriate level of expertise to confirm that it is of an acceptable scientific standard, however I have significant reservations, as outlined above.

# Response

**Published:** 2023-10-24

**Authors:** Asima Karim

**Affiliations:** Basic Medical Sciences, University of Sharjah, Sharjah, Sharjah, United Arab Emirates

We thank the reviewers for the favourable comments. We have addressed all the comments raised by the reviewer and highlighted the corrections throughout the revised manuscript. We believe that the reviewer's comments helped to significantly improve the quality of the manuscript. I am sharing with you the revised version of our manuscript entitled **" Time-related changes in the knowledge of HIV/AIDS among followers of various religions in India". **The point-by-point response to the reviewer’s comments is provided below.

**Reviewer # 1**

**General comments:**

 The study is interesting since HIV is one of the major public health concerns. Some revisions are required to improve the manuscript.

 Thank you

**Specific comments:**

**1.** **Introduction:**

 "The scarce literature on this topic involves a small cohort with indefinite conclusions, ". What does 'a small cohort' refer to?

**Author response: **It refers to the homogeneity of the samples across studies. As referenced in the manuscript, the majority of the studies examining HIV-related knowledge were conducted on the students (PMID: 34195038). This has been clarified in the revised manuscript.

**2.** **Methods:**

 Please explain, how the authors' approach to evaluate whether the differences in HIV knowledge increase were mainly due to the difference based on religions but not due to differences in socio-demographic characteristics. Can the authors add an analysis about this?

**Author response: **The current study presents the secondary analysis of cross-sectional surveys carried out in India during 2005-06, 2015-16, and 2019-21. The aim of the study was to investigate trends in the levels of knowledge of HIV/AIDS among Hindus, Muslims, and Christians in relation to standard socio-demographic variables over a period of 16 years. As the data was collected cross-sectionally it cannot measure causal relationships. Therefore, we believe that the addition of such analysis will change the focus of the paper and will be confusing for the reader.

**3.** **Results:**

 (A) It is stated that 'The development in fraction of the Indians who have heard about HIV/AIDS is presented in Figure 1'. However, Figure 1 displays about average HIV knowledge. Please clarify.

**Author response: **This has been corrected in the revised manuscript.

 (B) The discussions for Tables 3, 4, and 5 were mainly about the highest/lowest increase of HIV knowledge over a 16-year time period. If possible, please add a column providing the increase in HIV knowledge over this period in Tables 3, 4, and 5. Please also add subgroups based on religions in Table 3.

**Author response: **We thank the reviewer for this comment however, we would like to leave the tables as such, but of course, we are willing to reconsider if the reviewer believes that the suggested addition is really important for the story.

 (C) "The greatest increase in overall HIV knowledge was seen for Muslims in the bottom quintile, ...". Is it based on wealth? If yes, please add this information to the sentence to make it clearer for the readers.

**Author response: **Yes, now we have corrected it as per suggestion.

**4. Discussion:**

 (A) "We found that Christians had the highest increase in knowledge followed by Hindus, whereas Muslims had the least increase." Did the authors mean by 'the highest increase' is actually 'the highest level of HIV knowledge'. Please clarify.

**Author response: **Yes, that is largely correct. Since the manuscript is examining the time-related changes in HIV knowledge across Indian major religions, the sentence is phrased to reflect the change across time. However, if the recent dataset is considered cross-sectionally Christians had the highest percentage with correct HIV knowledge.

 (B) "We observed a clear tendency that the lower the initial HIV knowledge in 2005-06, the higher is the increase in HIV knowledge from the first to the last survey, which meant that differences in the level of HIV knowledge between different population groups had been reduced over time." Please discuss this finding including the implication for public health interventions.

**Author response:** This point is well taken. Now we have discussed this in the revised manuscript and three new references have also been added (Reference numbers 1, 2 and 3) in this context.

 (C) "Christians reported the highest increase in the level of HIV/AIDS-related knowledge" then what are the implications of this study to help in designing strategies for public health interventions related to different religions? And again, does 'the highest increase' refer to 'the highest level' of HIV knowledge?

**Author response: **We thank the reviewer for this comment. The implications of this finding have been incorporated in the revised manuscript accordingly. Yes, the highest increase refers to the highest level of HIV knowledge.

 (D) We found that men had a higher trend of increase in HIV-related knowledge as compared to females." However, in Christian, the HIV knowledge of men increased from 52.5 to 74.4 (an increase of 21.9), while women from 47.8 to 71.2 (an increase of 23.4). The same cases for Muslims and Hindus. Please clarify.

**Author response: **Yes, females had a higher increase in HIV-related knowledge. We apologize for the mistake and it has now been corrected in the revised manuscript and a new reference # 4 has been cited to support the findings.
